# Quality-by-Design-Based Development of n-Propyl-Gallate-Loaded Hyaluronic-Acid-Coated Liposomes for Intranasal Administration

**DOI:** 10.3390/molecules26051429

**Published:** 2021-03-06

**Authors:** Fakhara Sabir, Gábor Katona, Edina Pallagi, Dorina Gabriella Dobó, Hussein Akel, Dániel Berkesi, Zoltán Kónya, Ildikó Csóka

**Affiliations:** 1 Faculty of Pharmacy, Institute of Pharmaceutical Technology and Regulatory Affairs, University of Szeged, H-6720 Szeged, Hungary; fakhra.sabir@gmail.com (F.S.); katona.gabor@szte.hu (G.K.); edina.pallagi@pharm.u-szeged.hu (E.P.); dobo.dorina@pharm.u-szeged.hu (D.G.D.); hussein.akel@hotmail.com (H.A.); 2 Faculty of Science and Informatics, Department of Applied & Environmental Chemistry, University of Szeged, H-6720 Szeged, Hungary; daniel.berkesi@chem.u-szeged.hu (D.B.); konya@chem.u-szeged.hu (Z.K.)

**Keywords:** intranasal delivery, hyaluronic acid, scavenging assay, risk assessment, in vitro release study

## Abstract

The present study aimed to develop n-propyl gallate (PG)-encapsulated liposomes through a novel direct pouring method using the quality-by-design (QbD) approach. A further aim was to coat liposomes with hyaluronic acid (HA) to improve the stability of the formulation in nasal mucosa. The QbD method was used for the determination of critical quality attributes in the formulation of PG-loaded liposomes coated with HA. The optimized formulation was determined by applying the Box–Behnken design to investigate the effect of composition and process variables on particle size, polydispersity index (PDI), and zeta potential. Physiochemical characterization, in vitro release, and permeability tests, as well as accelerated stability studies, were performed with the optimized liposomal formulation. The optimized formulation resulted in 90 ± 3.6% encapsulation efficiency, 167.9 ± 3.5 nm average hydrodynamic diameter, 0.129 ± 0.002 PDI, and −33.9 ± 4.5 zeta potential. Coated liposomes showed significantly improved properties in 24 h in an in vitro release test (>60%), in vitro permeability measurement (420 μg/cm^2^) within 60 min, and also in accelerated stability studies compared to uncoated liposomes. A hydrogen-peroxide-scavenging assay showed improved stability of PG-containing liposomes. It can be concluded that the optimization of PG-encapsulated liposomes coated with HA has great potential for targeting several brain diseases.

## 1. Introduction

Intranasal delivery has been considered as one of most suitable alternative drug delivery routes to directly access the brain, bypassing the blood–brain barrier (BBB) [1,2,3]. This route of administration has the potential to deliver active pharmaceutical agents in higher concentrations in several brain diseases, including tumor and neurodegenerative diseases as Alzheimer’s and Parkinson’s diseases. In overcoming the difficulties of brain targeting arising from the physiological defense mechanism of the BBB, the quality-by-design (QbD) methodology offers a suitable tool to develop more appropriate and efficient carrier systems [2,4]. For that purpose, liposomes are considered as the most suitable carrier due to their increased bioavailability, scalability, and high stability [5,6]. The lipid carrier system is well tolerated through intranasal administration, and it can minimize cytotoxicity compared to other nanoparticles. Lipid nanoparticles have a wide range of particle sizes, from 50 to 1000 nm [7]. However, for nose-to-brain delivery via axonal transport, the particle size range between 50 and 700 nm is the most suitable [8].

**Figure 1:**
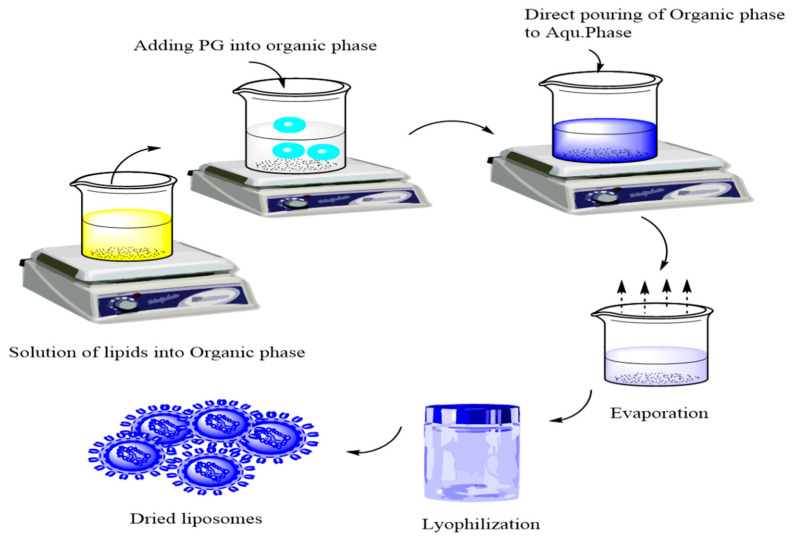
Direct pouring method for preparation of liposomes.

Hyaluronic acid (HA) is a naturally occurring biopolymer in living organisms. Its advantageous properties, including muco-adhesion, biocompatibility, and targetability to HA receptors (CD44), which are highly expressed in various tumor and stem cells, support intranasal application. The coating of liposomes with HA increases their negative zeta potential due to the addition of the negative carboxylate residue of HA on the surface of the liposomes, thereby increasing directly their stability [9]. A novel bottom-up method of preparation called the direct pouring method (DPM) is a modified form of the solvent injection and emulsification method but quite feasible as it avoids the use of extra size reduction, extrusion, or filtration techniques. DPM involves two simple steps, the solubilization of lipids into the organic phase and the direct pouring of the organic phase into the aqueous phase along with the evaporation of the organic solvents, as shown in Figure 1.

n-Propyl gallate (PG), also known as propyl 3,4,5-tri-hydroxybenzoate, an ester form of gallic acid and propanol, functions as a synthetic antioxidant and preservative, can inhibit nucleic acid synthesis in micro-organisms, and prevent their growth [10]. PG can be promising in the treatment of brain tumors, e.g., glioblastoma multiforme, as long as it can reach the brain. Alternative administration routes are preferred as previously a slight toxic effect was reported in the case of oral administration [11]. For that purpose, the intranasal route can be effective. PG’s nose-to-brain applicability is supported by its low molecular weight (212.2 g/mol), lipophilic character (logP = 1.8), and pKa (7.94); as a result, the non-ionized fraction of the drug dominates in nasal conditions (pH = 5.5–6.5), enhancing absorption. However, PG is particularly insoluble in water, and it is sensitive to the nasal environment because of its hydrogen donor nature. Therefore, suitable carriers, such as liposomes, are required to enhance its stability in and affinity for biological membranes and, moreover, to increase its concentration at the target site, eliminating local irritation [12]. Previous studies showed that PG may contribute to mitochondrial impairment and the inhibition of cellular respiration. PG has proven efficacy and has vital anti-cancer effects on various cancer cells that may lead to DNA genotoxicity, cytotoxicity, and fragmentation [13]. It also has been revealed that PG used with other antitumor agents in various in vivo studies exhibited significant efficacy in gastrointestinal tract cancer. The anti-cancer mechanism of PG is based on inhibition of the cell growth cycle via the production of reactive oxygen species and the stimulation of the autophagy of malignant cells [10,14].

The QbD concept as a risk-and-knowledge-based quality management method aims to achieve the desired product quality and fulfill the therapeutic need. The data from previous studies have revealed that this approach can be applied during all formulation development stages [15,16,17]. The QbD-based formulation strategy involves the following stages: (1) defining and identifying the quality target product profile (QTPP) based on the past literature and appropriate bioinformatics relevance, (2) defining the product design and development process according to a predefined product profile, (3) identifying potential critical quality attributes (CQAs) and critical process parameters (CPPs) and performing a risk assessment (RA), (4) setting up with the help of RA results practical development (the design-of-experiments step of the method) and implementing it in accordance with the most relevant factors (CPPs, CQAs) selected by RA to define the design space, (5) developing a process control strategy to ensure persistent product quality, and (6) managing the product quality during its shelf life [18,19].

As stated by International Conference for Harmonisation (ICH) guidelines, the development of novel drug delivery systems should be supervised heavily under quality control methods, for which QbD provides a suitable tool [20,21,22]. In the case of liposomal formulations, there are many risk factors that should be taken into account, including particle characteristics, structure, dissolution and permeability profiles, and the production procedure as well. With the help of QbD, the product can meet the criteria of liposomal formulations, the nose-to-brain delivery pathway, and the enhanced therapeutic effect. The main advantage of using QbD lies in the fact that it is a continuous feedback system that could eliminate the possibility of errors at an early stage of production. With early set criteria, proper particle characteristics with optimized size and size distribution along with a zeta potential value of PG-loaded liposomes can be ensured, representing high stability in colloidal solution form. Besides, the structure of the final product in solid form is also crucial in connection with solubility, particularly its dissolution and absorption profile. Qbd-driven optimization of liposomes for the nasal administration route has already been successfully applied in a previous study [23].

This study focused on the QbD-based development of PG-loaded liposomes coated with HA for intranasal delivery. The coating of liposomes with hyaluronic acid can improve their stability by reducing macrophage uptake after absorption from the nasal mucosa. Moreover, it can enhance the targetability of the nanocarrier. In addition, the use of QbD strengthens the validation of the novel method (DPM) for the development of HA-coated liposomes [18,24,25].

## 2. Results

### 2.1. Initial Knowledge Space Development

**Figure 2:**
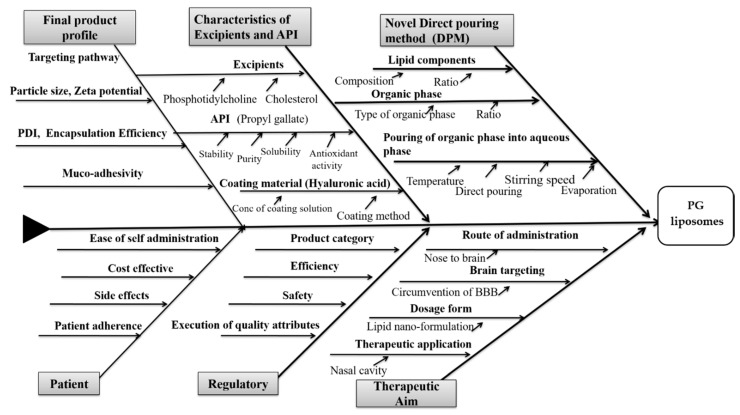
Ishikawa diagram showing the cause-and-effect relationships between influencing factors of hyaluronic acid (HA)-coated, n-propyl gallate (PG)-loaded liposomes for nose-to-brain drug delivery.

Several different factors could have a significant impact on the quality of a PG-containing liposomal formulation for nasal administration (Figure 2).

The collected factors presented in the Ishikawa diagram include all the possible critical factors affecting the final quality of the coated liposomal formulation. It was developed following the recent scientific data regarding coating and nose-to-brain delivery and helps in recognizing the cause-and-effect relationship between the aimed product and its influencing factors. Based on the fishbone diagram, QTPPs can be selected. These selected parameters with their targets and their justification are listed in Table 1.

The present study considered the following CQAs, depending upon the predefined goals and therapeutic needs: excipients (phospholipids, cholesterol as its quality may affect the end product profile), coating material, Z-average, zeta potential, polydispersity index (PDI), and aqueous phase. All these factors are relevant to the quality of the product [24,31]. The process variables of the following new approach included the sonication time (for mixing lipids into the organic phase), temperature (for evaporation), and rotations per minute (rpm) of the hot plate (speed of rotation for evaporation).

### 2.2. Results of Risk Assessment

**Figure 3:**
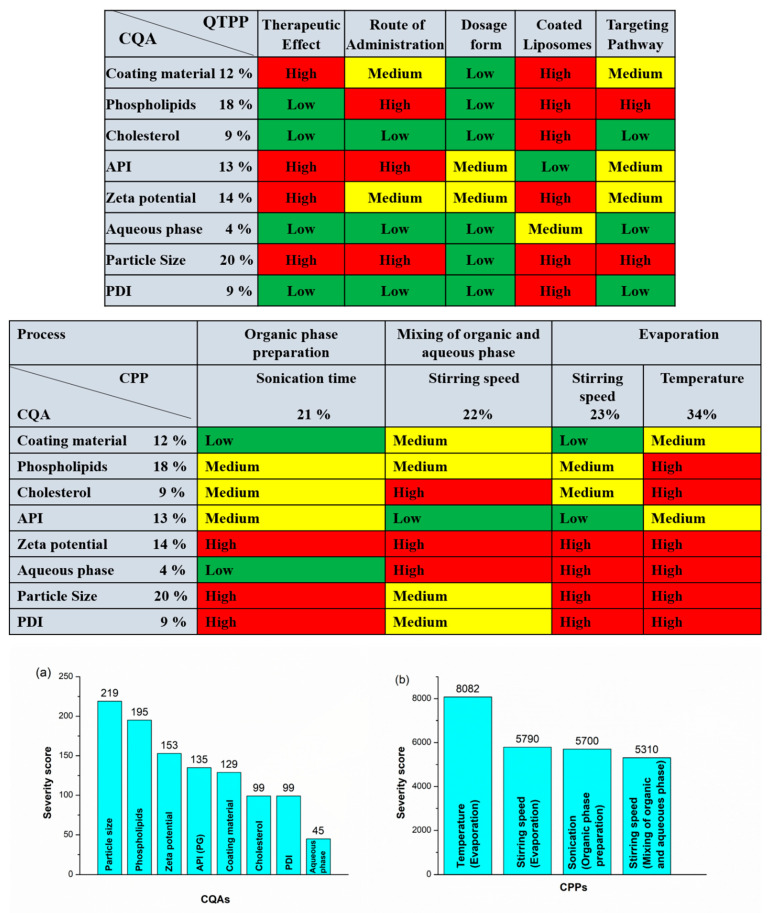
Interdependence rating and estimation of QTPP elements and critical quality attributes (CQAs) and of CQAs and critical process parameters (CPPs), and Pareto charts showing the severity/impact among selected (**a**) CQAs and (**b**) CPPs.

After determination of the impact (H, M, L) of the QTPPs and evaluation of the CPPs and CQAs, RA was applied in order to follow the QbD-based formulation design and development. For RA, an estimation of the interdependence between the QTPP elements and CQAs and between the CQAs and critical material attributes (CMAs/CPPs) was made first by using the previously mentioned 3-grade scale, as their impact on each other is high, medium, or low. The assessment of the level of effect depends on the scientific knowledge in the literature and experimental experience. The occurrence of the CPPs was also estimated. Based on the observations, the severity or impact score for each factor determining the final liposomal product was calculated. The severity scores and their order are shown in Pareto charts generated by the software. In this case, these scores for every CQA and CPP are shown in Pareto charts in Figure 3. The Pareto chart principle known as the 80/20 rule describes that for many events, 80% of the effects come from 20% of the causes. These 20% of critical factors require 80% of focused measurement in the drug development process. The rankings presented in the charts of Figure 3 helps to create a priority list by the factors’ having critical effect on the aimed end product and describe the influencing properties of each factor. The order of the CQAs is shown (Figure 3a); the quantity of phospholipids, cholesterol, and active pharmaceutical ingredients (APIs) has the same and highest critical effect on the required quality of the final product. A lower critical effect is exerted on product quality by the rest of the factors in the diagram: the coating material, zeta potential, and aqueous phase. Figure 3b shows the rankings of the CPPs related to the present liposome development process. It shows that the temperature of the DPM has the greatest critical effect on the final product. After temperature, the rotation speed for both mixing and evaporation has been shown as having an almost equal effect on product quality, while the severity score is the lowest for mixing sonication time, indicating that this factor has the least effect on the final quality of the end product. Depending on the result of the previously presented initial RA, the factors for the Box–Behnken design (BBD) of the experiment could be selected.

### 2.3. Design of Experiments: Box–Behnken Design

The RA results helped to align the experimental design. The critical factors with the highest severity scores were selected to screen the effects of the formulation parameters on the quality of the final coated liposomal formulation. These were the following: the quantity of phospholipids (L-α-phosphatidylcholine (PC)) and the quantity of cholesterol (mg) selected from the CQAs, and the temperature (°C) of evaporation taken from the CPPs as variable X_2_. According to our selection methodology, the Z-average, PDI, and zeta potential were selected as dependent factors. The highly significant parameters were selected after applying QbD and subjected to screening with respect to the obtained optimized formula for all studied variables within accepted ranges. BBD screening was validated by ANOVA for each factor (ANOVA table obtained from the design of experiments (DOE) results). Table 2 shows all the values of responses for 15 runs of BBD.

### 2.4. Influence of Investigated Parameters on the Z-Average, PDI, and Zeta Potential

**Figure 4:**
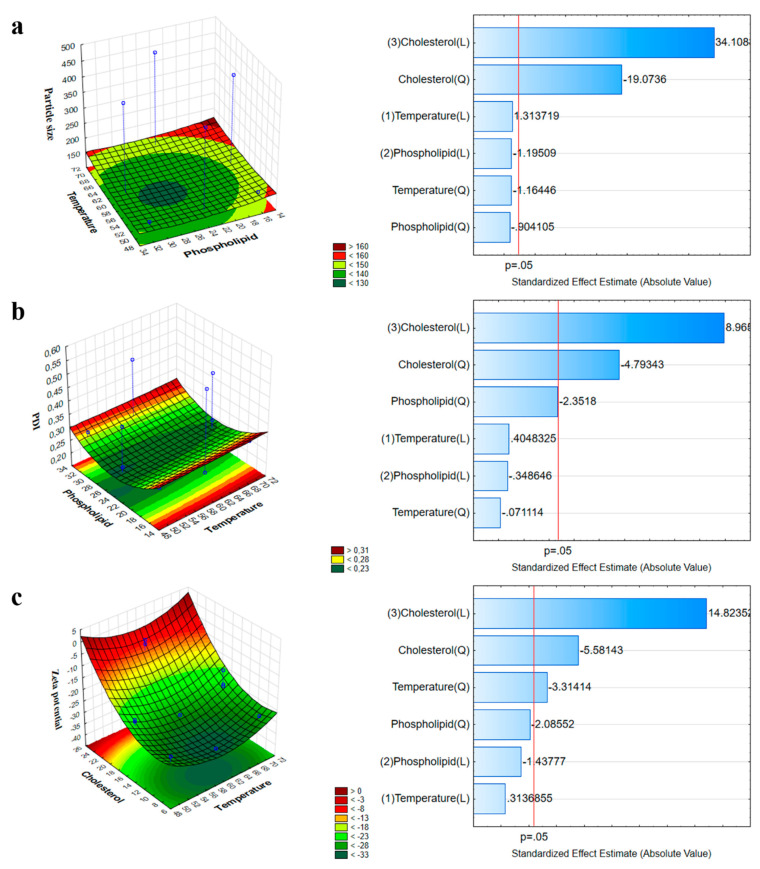
Three-dimensional surface plot and Pareto chart showing the combined impact of phospholipids and temperature on the Z-average (**a**), on the PDI (**b**), and on the zeta potential (**c**).

The result of the significance of effects for each variable explained the interdependence between the studied independent and dependent responses. In colloidal drug delivery systems, the Z-average is closely related to the particle size; therefore, a change in it can indicate changes in the primary particle size as well. Based on the BBD results, an optimized colloidal stable system was obtained. At a lower concentration of PC, a higher Z-average was obtained, but as the concentration of the phospholipids was increased up to a significant amount, the Z-average decreased to 130 nm. A further increase in the amount of phospholipids led to the formation of particles of large sizes, as shown in Figure 4a. Phospholipids act as a surfactant and result in higher solubility at the interface of two phases, leading to a reduced Z-average. However, as the concentration of phospholipids increased, due to higher viscosity, the Z-average increased. The effect of temperature was similar; at a lower temperature, a larger Z-average was observed, but its size decreased with an increase in temperature during the process. The surface polynomial (Equation (1)) obtained for the fitted full model explaining the effect of formulation and process variables on the mean Z-average is:

(1) $$Z-average=231.82+11.9x_{1}-7.239x_{2}+301.73x_{3}-4.88x_{1}x_{2}+24.88x_{1}x_{3}-5.52x_{2}x_{3}-9.87x_{1}^{2}-8.7x_{2}^{2}-136.2x_{3}^{2}$$

with R² = 0.998, adjusted R² = 0.996, and mean square (MS) = 85.858.

The influence of phospholipids, cholesterol, and temperature on the PDI was also investigated (Figure 4b).

At average or moderate values of these three factors, the PDI value fell within the optimum range, while at the two extremities, the PDI value was high. According to the following results, only cholesterol had a significant effect on the variation in the PDI.

The polynomial equation (Equation (2)) obtained for the fitted full model demonstrating the effect of formulation variables on the PDI is:

(2) $$PDI=0.334+0.0089x_{1}+0.0015x_{2}+0.2233x_{3}-0.0194x_{1}x_{2}+0.049x_{1}x_{3}-0.0531x_{2}x_{3}+0.0089x_{1}^{2}-0.0513x_{2}^{2}-0.1013x_{3}^{2}$$

with R² = 0.969, adjusted R² = 0.901, and MS = 0.001.

The effect of phospholipids, cholesterol, and temperature on the zeta potential was also studied and is shown in the corresponding coefficient obtained from the DOE (Figure 4c). The coefficient showed that temperature and cholesterol had a significant effect on the stability of liposomes as compared to the concentration of phospholipids. 

The polynomial equation (Equation (3)) obtained for the fitted full model demonstrating the influence of formulation variables on the zeta potential is:

(3) $$Zeta\;potential=19.6+0.491x_{1}-1.67x_{2}+22.42x_{3}-2.71x_{1}x_{2}+1.216x_{1}x_{3}-2.141x_{2}x_{3}-4.43x_{1}^{2}-2.76x_{2}^{2}-7.016x_{3}^{2}$$

with R² = 0.972, adjusted R² = 0.948, and MS = 4.508.

The surface plot shows that the zeta potential of the formulation is significantly dependent on the applied amount of phospholipids and the process temperature. The formulation of liposomes with a low concentration of cholesterol at 60 °C resulted in a stable lowest surface charge and, therefore, increased stability.

### 2.5. Characterization of Z-Average, PDI, and Zeta Potential and Analysis of Coated and Uncoated Liposomes

The Z-average, PDI, and zeta potential were measured both for optimized PG-loaded uncoated and HA-coated liposomes. The Z-average of uncoated liposomes was reported as 135.2 ± 5.2 nm, with a PDI of 0.094 ± 0.001 and a zeta potential of −29.9 ± 5.8 mV, and of coated liposomes was found to be 167.9 ± 3.5 nm, with a PDI of 0.129 ± 0.002 and a zeta potential of −33.6 ± 4.5 mV (Table 3.). All the results met the standard set criteria, as mentioned above. The Z-average range was between 123 ± 2.5 nm and 300 ± 3.3 nm. The particle size of uncoated liposomes was lower, as mentioned in the previous section, than that of coated ones, which showed that the HA coating of liposomes was successful. The PDI was lower for optimized liposomes, which indicated a more uniform vesicle size for the final formulation. The zeta potential of coated liposomes was higher than that of uncoated liposomes, which showed increased stability of coated liposomes.

### 2.6. Determination of Acetone Residual in the Formulations

As acetone belongs to Class 3 solvents, its residual concentration should be less than 5000 ppm in the daily dose of the final product, a requirement of the International Conference for Harmonisation (ICH) Q3C (R5) guideline for residual solvents. The residual acetone content was determined in both coated and uncoated formulations using gas chromatography, shown in Table 4.

Both results were high under the standard criteria of maximum allowed residual level, supporting the applicability of the DPM for the preparation of liposomes.

### 2.7. Encapsulation Efficiency, Percentage Yield, and Drug-Loading Analysis

The encapsulation efficiency was found to be 90 ± 3.6% and showed very good results for PG-loaded liposomes. The percentage yield was 71.33 ± 1.52%, which also indicates that the results of PG-loaded liposomes were within the aimed criteria. The drug-loading capacity of the liposomes was 2.81%, which can be associated with a higher amount of lipid applied for the wall-forming agent. The measured encapsulation efficiency (EE) of the optimized formulation was higher, with the purpose of having a high amount of the drug at the site of action [32].

### 2.8. Fourier-Transform Infrared Spectroscopy (FTIR) Spectroscopy Analysis

**Figure 5:**
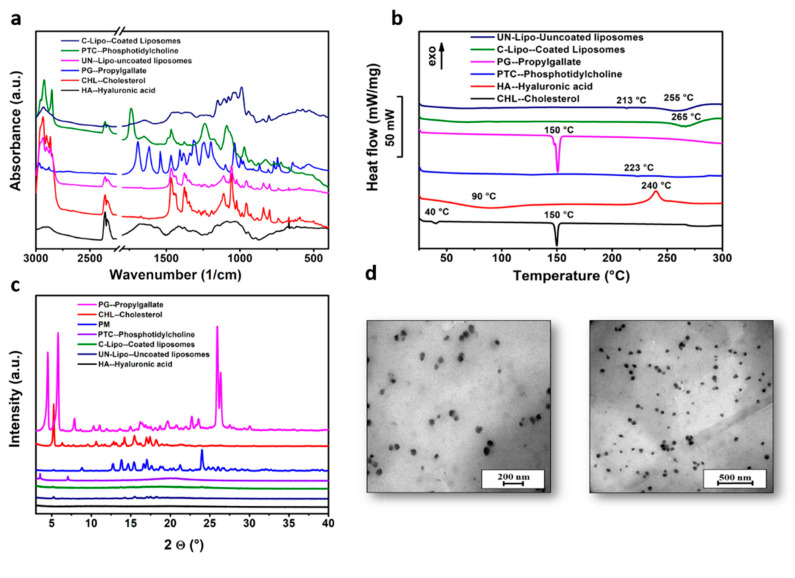
Fourier-transform infrared spectroscopy (FTIR) spectra (**a**), differential scanning calorimetry (DSC) thermograms (**b**), and X-ray powder diffraction (XRPD) diffractograms of coated and uncoated liposomes and components of liposomal formulation (**c**), and transmission electron microscopy (TEM) images of liposomes at different resolution scales (**d**).

The FTIR spectra of the components of coated and uncoated liposomes are presented in Figure 5a. Each spectrum includes two different regions. The high-wave-number part of the spectrum (3000–2800 1/cm) derives from C-H stretching vibrations only. In turn, it mainly originates from the hydrocarbon chains. The low-wave-number region of the spectrum (below 1800 1/cm) shows the fingerprint region. The ester ν (C=O) is usually the strongest peak, followed by the phosphate contributions near 1240 1/cm (ν_as_(PO^2−^)) and 1090 1/cm (ν_s_(PO^2−^)). The peaks at ~900–600 1/cm are essentially coherent to the polar head groups of the lipids. The hydrocarbon chains do contribute near 1465 1/cm, but all-trans conformations absorb near 1470 1/cm, where there is C–H deformation of CH_2_. The contribution of the lipid hydrocarbon chains is present in various spectral regions (3000–2800 1/cm). This region contains C-H stretching bands from different vibrational modes: ν_as_ (CH_2_) near 2920 1/cm, ν_s_ (CH_2_) near 2850 1/cm, ν_as_ (CH_3_) near 2960 1/cm, and ν_s_(CH_3_) near 2870 1/cm. Compatibility study results showed that there was no interaction between the formulation components. The spectra of coated liposomes showed peaks at ~1650 1/cm (amide I of the α-helical region) and 1050 1/cm (C-O-C stretching vibrations of the α-glucopyranose structure), that could not be observed in the spectra of uncoated liposomes, suggesting that the HA coating was successfully performed.

### 2.9. Differential Scanning Calorimetry (DSC) Analysis Results

The thermal behavior of PG, cholesterol, PC, HA, and liposomes coated and uncoated with HA was investigated with DSC (Figure 5b). The endothermic peaks at 150 °C on the DSC curves of PG and cholesterol correspond to their melting points. PC and HA did not exhibit any distinct melting event, possibly due to their non-crystalline nature, while an endothermic peak at 223 °C (PC) and an exothermic peak at 240 °C (HA) was observed due to their degradation pattern. The decomposition of HA can also be observed on the DSC curves of liposomes at 255 °C (uncoated) and 265 °C (coated), but the decomposition of PC as a wall-forming agent was only detected in the case of uncoated liposomes at 213 °C. HA coating increased the thermal stability of liposomes and prevented them from degradation until 265 °C. Since the thermograms of PG-loaded liposomes did not display any endothermic peaks at 150 °C compared to the pure PG thermogram, the encapsulation of PG in the liposomes was indicated. 

### 2.10. X-ray Powder Diffraction (XRPD) Analysis Results

XRPD studies showed the crystalline nature of PG and cholesterol and the amorphous nature of PC and HA as coating materials in accordance with the DSC results, as shown in Figure 5c. In the diffractograms of uncoated and coated liposomes, the characteristic peaks of cholesterol (at 5.18, 13.8, 15.34, 16.6, 17, and 17.6 2θ) as a wall-forming agent can be observed, but in the case of coated liposomes, these peaks disappeared. The characteristic peaks of PG could be detected in the liposomal formulations, which shows that the drug was encapsulated.

### 2.11. Morphology of Liposomes

The transmission electron microscopy (TEM) images of PG-loaded liposomes with coating and without coating showed the spherical shape and uniform distribution of liposomes, as shown in Figure 5d, showing a monodisperse distribution without any aggregation. The TEM images revealed that particle sizes were ~100–120 nm. The morphological study via TEM revealed that particle sizes were ~100–120 nm. The results of TEM images were comparable with the dynamic light scattering (DLS) results and were within the nano-range (the Z-average values were ~135 2 ± 5.2 and 167 ± 3.5 nm), underlining that the acquired Z-average results are suitable for explaining the effects of synthesis parameters on particle size. 

### 2.12. In Vitro Release Studies

**Figure 6:**
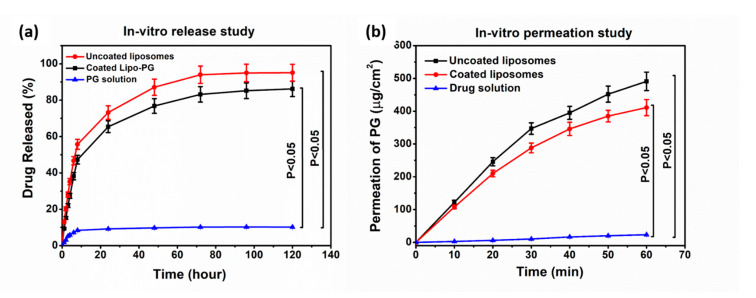
In vitro release study of PG in phosphate buffer (pH 5.6) (**a**) and in vitro permeability studies of uncoated-liposomes, coated liposomes, and PG solution (**b**).

In vitro release studies of uncoated and HA-coated liposomes showed 75% and 60% drug release within 24 h, respectively, in simulated nasal electrolyte solution (SNES) (pH 5.6), as shown in Figure 6a, while 80% of the drug was released within 48 h from coated liposomes. The release of PG from solution was significantly lower (10% within 24 h) in comparison to other liposomal formulations. Drug release kinetics were determined by fitting kinetic models, and data were evaluated by the correlation coefficient (R^2^) (Table 5). R^2^ values were used as a standard to choose the best model to describe drug release from the liposomes and the drug solution. In the case of uncoated and HA-coated liposomes, the R^2^ obtained for fitting the release data of PG to the Higuchi model indicated that drug release from the liposomes was diffusion controlled, as shown in Table 5. In vitro release study results have shown that the slower release from coated liposomes is due to the restriction of water diffusion into the carrier matrix, which subsequently slows down the drug release rate. Another possible reason is that coating adds a barrier against drug diffusion with regard to the electrostatic interaction between the positively charged PC and the negatively charged HA [33]. The kinetic model data evaluated the release of PG following the diffusion-controlled release, as shown in Table 5.

### 2.13. In Vitro Permeability Study

The modified side-by-side-type apparatus was used for the in vitro nasal permeation study of uncoated and coated PG-loaded liposomes and the PG solution. Figure 6b shows the rate of PG permeation from the donor to the acceptor phase. The maximum permeation of PG from coated liposomes that was measured after 60 min was 420 μg/cm^2^. The PG-containing uncoated liposomes provided faster diffusion and higher drug concentration (>500 μg/cm^2^ after 60 min) in comparison to the PG solution. In the case of the PG solution, permeation was negligible, about 5 μg/cm^2^. However, when compared with coated liposomes, there was a difference of 80 μg/cm^2^ between the permeation values. A slightly lower permeation rate of coated liposomes was observed due to the muco-adhesive nature of HA, which helps to increase the residence time of the coated formulation on the mucosa and protects it from premature mucocililary clearance. The in vitro permeation of the PG-containing coated liposomes provided faster diffusion and higher drug concentration in comparison to the PG solution [34].

### 2.14. Antioxidant Activity Measurement with Hydrogen Peroxide (H_2_O_2_)-Scavenging Assay

**Figure 7:**
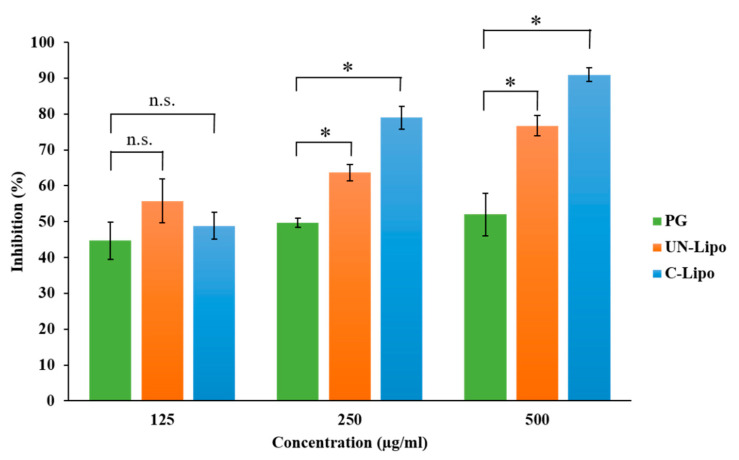
Percentage inhibition of hydrogen-peroxide-scavenging activity of different concentrations of PG-containing coated and uncoated liposomes in comparison to the initial PG solution. Statistical analysis: t-test. * *p* < 0.05; n.s. means not significant compared to the PG control.

The antioxidant activity of PG was investigated in order to confirm whether the DPM is gentle enough to encapsulate the drug in liposomes and to preserve its stability. Preserving enzyme activity is essential in the pharmacological effect. It has been demonstrated that free radicals assume an important role in the pathogenesis of specific diseases and also in different types of cancer. Coated and uncoated liposomes containing PG in different concentrations were screened for in vitro scavenging activity using hydrogen peroxide. The scavenging activity of the formulations and the initial PG solution (as a control) are presented in Figure 7. It was revealed that the liposomal formulations preserved the antioxidant activity of PG based on the scavenging activity measurement against H_2_O_2_. The hydrogen-donating activity, measured using hydrogen peroxide radicals as the hydrogen acceptor, demonstrated a strong association between the concentration of PG and the rate of inhibition. Increasing the concentration of PG enhanced the inhibition. At a low concentration of 125 μg/mL, no significant difference in the antioxidant activity was observed for both PG liposomes and the initial PG solution. In the case of higher PG concentrations (250 and 500 μg/mL), a significantly improved antioxidant activity of both coated and uncoated liposomes was reached, which can be explained by the stabilizing effect of the liposome carrier at a 95% confidence interval. The enzyme activity was remarkably higher in the case of coated liposomes, which proves that the HA coating enhances the stability of PG in comparison to uncoated liposomes. 

### 2.15. Accelerated Stability Studies

An accelerated stability test was carried out to investigate the physical stability and shelf life of both coated and uncoated formulations. Data were evaluated for the Z-average, EE, PDI, and zeta potential and during 6 months of storage, as illustrated in Table 6. The liposomes showed enhanced physical stability under accelerated stability study conditions, and no significant difference was observed between all measured physical parameters after the sixth month of the study period. Accelerated stability studies revealed that coated liposomes were more stable than uncoated liposomes, which suggests applying the coated carrier for therapy. The physical stability study validated the method of preparation (DPM) of both formulations, strengthening the evidence. The chemical stability of the drug was investigated retrospectively, the drug content was determined after 2, 5, and 8 months of storage, and the shelf life (t_90%_) was extrapolated. Experimental data showed that the shelf life of both formulations was lower than 2 months at 40 °C and 75% (RH), which suggests cold place storage (2–8 °C) of formulations to protect PG from degradation.

## 3. Discussion

Nose-to-brain delivery is among the most significant administration routes to target the brain. Lipid nanoparticles are a most suitable delivery system due to high biocompatibility, safety, stability, and sustained release [35]. In the present work, liposomes were prepared through the novel DPM and loaded with an antioxidant (n-propyl gallate). The QbD methodology and risk assessment strategy was used to develop optimized formulation via the DPM. Applying the novel DPM avoids the use of any additional size reduction or extrusion technique, which makes the preparation process more feasible and precise and less time consuming. Moreover, this method also provides a greater percentage yield of liposomes. This method has a profound similarity to the solvent injection method, but here, we also avoid slow injection using a syringe or another instrument to mix both aqueous and organic phases [36,37]. The authors recommend this technique for industrial scale-up for more optimum preparation of liposomes. The conventional techniques for liposome development and size are convenient to use with sophisticated equipment. However, problems related to scale-up and scale-down applications have motivated improvements to conventional processes that have also led to the development of novel methods for liposome formation. Based on the impact of process parameters, critical factors were determined. The critical factors with the highest severity scores were used in the three-factor BBD. After combining the selected critical factors, i.e., particle size, PDI, and zeta potential, the optimized formulation was obtained. Characterization studies showed that the Z-average of uncoated liposomes was 135.2 ± 5.2 nm, with a PDI of 0.094 ± 0.001 and a zeta potential of −29.9 ± 5.8 mV, and for coated liposomes was 167.9 ± 3.5 nm, with a PDI of 0.129 ± 0.002 and a zeta potential of −33.6 ± 4.5 mV, with a 90 ± 3.6% EE. Compatibility study results showed no interaction between formulation components. FTIR measurement confirmed in the case of coated liposomes peaks appeared at ~1650 1/cm (amide I of the α-helical region) and 1050 1/cm (C-O-C stretching vibrations of the α-glucopyranose structure), suggesting that the HA coating of liposome was successful [38]. The in vitro release study results showed differences between coated and uncoated liposomes. The reason for the slower release from coated liposomes is the restriction of water diffusion into the carrier matrix, which subsequently decreases the drug release rate. Another possible reason is that coating adds a barrier against drug diffusion with regard to the electrostatic interaction between the positively charged L-α PC and the negatively charged HA [39]. The kinetic models data evaluated the release of PG following the Highuchi model of release [33]. The in vitro permeation of the PG-containing coated liposomes provided faster diffusion and higher drug concentration in comparison to the PG solution. In the case of the PG solution, the permeation was negligible, about 5 μg/cm^2^. However, when compared with uncoated liposomes, there was a clear difference between both permeation values. The lower permeation rate of coated liposomes is due to the muco-adhesive nature of HA, which helps to increase the residence time of the coated formulation in the nasal mucosa and protects it from premature mucociliary clearance. The anti-cancer activity of PG is based on the removal of free radicals. The most important mechanism to achieve this goal is the donation of hydrogen to free radicals to convert them to nonreactive species. The donation of hydrogen would remove the odd electron that is responsible for radical reactivity. In this study, for the evaluation of antioxidant activity, a hydrogen peroxide (H_2_O_2_)-scavenging assay was performed. Results concluded a proportional antioxidant activity increase at higher concentrations of PG. The enzyme activity was remarkably higher in the case of coated liposomes, which proves the HA coating enhances the stability of PG in comparison to uncoated liposomes. The results ensure potential use of PG against brain tumor via the intranasal route. There are several studies in favor of in vivo outcomes regarding PG that have reported anti-cancer, antioxidant, and anti-angiogenic activity [40]. PG induces apoptosis in human leukemia cells and HeLa cells by increasing the reactive oxygen species (ROS) or glutathione depletion. PG also plays a significant role in autophagy, which, in turn, plays an important role in cellular physiological processes [41]. Autophagy is stimulated as a stress response to pathological conditions, including inflammation, starvation, and cancer. The literature suggests that the regulation of autophagy is complex, occurring via the Akt/mTOR and MAPK/Erk1/2 signaling pathways, and serves as a potential treatment against cancer [42]. Wei et al. studied the PG anti-cancer activity in hepatocellular carcinoma cell growth via the induction of reactive oxygen species and the activation of autophagy. The studies found that PG also enhances the intracelular levels of superoxide and reactive oxidative stress, which could result in autophagosomes and lysosomes [13,43]. The accelerated stability studies were performed over a period of 6 months. All the measured parameters for stability studies, including the particle size, EE, PDI, and zeta potential, revealed that coated liposomes are more stable than the uncoated liposomes.

## 4. Materials and Methods

### 4.1. Materials

PG purchased from Sigma-Aldrich (Budapest, Hungary) was applied as the antioxidant drug. The excipients HA (Mw = 1400 kDa) (Sigma-Aldrich, Budapest, Hungary), L-α-phosphatidylcholine (PC) from soybean (Sigma-Aldrich, Budapest, Hungary), cholesterol (Molar Chemicals Ltd., Budapest, Hungary), 96% ethanol (Molar Chemicals Ltd., Budapest, Hungary), and sodium chloride physiological solution (Molar Chemicals Ltd., Budapest, Hungary) were used in the formulation.

### 4.2. Initial Knowledge Space Development and Collection of Influencing Factors of PG-Lipo

The QTPP is defined as the potential quality attributes of a product that will be achieved to ensure the required characteristics, considering the safety and efficacy of the final product. It is the basis of structuring product design and development that could be achieved, which includes product performance that meets the regulatory-based professional need as well as patient and clinical application related to product performance. The screening of the QTPP from the previous literature was done with precise planning and contemplation based on the relevant International Conference for Harmonisation (ICH) guidelines [17,44]. The QTPP selection is the initial step of the QbD methodology used in this research, and to set up the criteria for this screening, a prospective summary of the literature and the experience from previous studies are required. This is called knowledge space development and includes materials, techniques, and other formulation components. The Ishikawa diagram as a quality management tool was applied for evaluating the cause-and-effect relationships for screening the CQAs that influence the final product profile [45]. This cause-and-effect diagram is also significant for the selection of the QTPP and CPPs in new QbD-guided design development [25,46]. The identification and selection of factors having critical effects on the product is the second step of the QbD approach. These factors are called CQAs, which have a critical influence on the final product quality and can be evaluated or controlled. These are basically biological or microbiological, physical, or chemical; CQAs should be in the appropriate range or limit to ensure the final product quality characteristics. The list of CQAs is always unique; it depends on the QTPP as defined initially. In this step of the QbD approach, critical process and/or material parameters should be analyzed. These factors or parameters are particularly relevant to the method used for the development of liposomes; hence, they may influence the CQAs.

### 4.3. Risk Assessment (RA)

After determining the QTPP and selecting the critical factors (CQAs, CPPs), the next step is to perform the RA. The RA is the evaluation of interdependence between the QTPP elements and CQAs and between the CPPs and CQAs. The RA was implemented by using Lean QbD^®^ software (QbDworks.com, QbDworksLLc., Fremont, CA, USA). The evaluation of connections between these screened factors was structured by using a 3-level scale. Each factor and parameter was thoroughly evaluated. The 3-grade scale reflected the impact of factor interactions with each other evaluated one by one in pairs, their interaction being low (L, green), medium (M, yellow), and high (H, red). This interdependence rating step was followed by an occurrence rating estimation of the CPPs using the same 3-grade scale. The determination of this risk occurrence is compulsory for analysis; hence it was performed using risk management protocols. As a result of the RA, the ranking of CQAs and CPPs was plotted on Pareto diagrams generated by the QbD software. The following Pareto chart ranked the impact of potential factors and highlighted the significant factors. This software application in the development of pharmaceutical formulation helps to select the items of the factorial design of experiments to obtain the optimized formulation strategy [46].

### 4.4. Design of Experiments by the Box–Behnken Design (BBD) for Optimizing the Composition of Coated Liposomes (C-Lipo)

Among the various methods of optimization, the BBD is the most broadly accepted and extrapolated in the design of the experimental phase of pharmaceutical formulation development. The significance of the BBD in the development of pharmaceutical formulations is based on screening and evaluating highly influential factors by applying the response surface methodology (RSM) with high precision and only a few experimental trials [46]. The 3-level BBD was exercised and carried out using TIBCO Statistica^®^ 13.4 software (Statsoft Hungary, Budapest, Hungary), and analysis of variance (ANOVA) was applied to calculate the statistical significance of each model coefficient at a 95% confidence level. Differences were considered significant when *p* < 0.05. For the factorial design, the variables were selected based on the RA results. By exercising the response surface methodology, three different independent factors and dependent factors were assessed: the independent factors included the amount of phospholipids (X_1_, 12 to 36 mg), the amount of cholesterol (X_2_, 8 to 24 mg), and the effect of temperature (X_3_, 40 to 80 °C), while the dependent factors included the Z-average (Y_1_), zeta potential (Y_2_), and polydispersity index (PDI) (Y_3_). The type and ratio of the organic solvent were constant. Polynomial equations showing the correlation among the independent and dependent variables were generated in order to get the optimized particle size, PDI, and zeta potential (i.e., the minimum Z-average and PDI and the optimum zeta potential).

### 4.5. Preparation of Liposomes via the DPM and Coating of the Lyophilized NPs with HA Polymer Solution

Liposomes were prepared using a novel direct pouring method. This method is a simple bottom-up size reduction technique and results in a stabilized formulation using the optimized concentration of lipids. Different amounts of PG (12, 20, and 40 mg), PC (12, 24, and 32 mg), and cholesterol (8, 16, and 24 mg) were dissolved in 4 mL of an ethanol:acetone (3:1) mixture and directly added to the 10 mL aqueous phase. The organic phase was evaporated at 60 °C under constant stirring (400 rpm) using a hot-plate magnetic stirrer. The unentrapped drug was removed by dialysis at 4 °C for further analysis. The liposome formulations were centrifuged for 1.5 h at 13,500 rpm to collect and separate the pellets from impurities and then redispersed in 1 mL of purified water. HA coating solution (50 mg/mL) was prepared by soaking HA on the surface of purified water under constant stirring at 400 rpm for 30 min until complete swelling occurred. Then 1 mL of HA solution was added dropwise by a syringe to the liposomal solution for 30 min under constant stirring at 700 rpm at room temperature. Thereafter, the mixture was stored at 5 ± 3 °C for 24 h. After the coating process, the liposomal formulation was centrifuged again at 4 °C at 25,000 rpm for 10 min to remove the free HA that did not go through the electrostatic coating of liposomes, and the pellet was redispersed in 10 mL of purified water. Then 1.5 mL of the coated formulation was transferred to vials and freeze-dried at −40 °C for 12 h under a 0.013 mbar pressure and kept at 25 °C for 3 h for secondary drying to obtain lyophilized powders using a Scanvac Cool safe laboratory freeze-dryer (Labogene, Lynge, Denmark). The powder vials were stored at 5 ± 3 °C for further investigations, including compatibility and morphological characterization.

### 4.6. Average Hydrodynamic Diameter, Surface Charge, and Polydispersity Index

In 5 mL of purified water, 5 mg of lyophilized liposomes was redispersed, and to reduce the inter-particle aggregation, the solution was sonicated for 5 min. The average hydrodynamic diameter (Z-average), surface charge (zeta potential), and polydispersity index (PDI) of the liposomes were analyzed in folded capillary cells using the Malvern nano ZS instrument (Malvern Instruments, Worcestershire, UK). The temperature and refractive index of the apparatus were set at 25 °C and 1.445, respectively, and the total number of scans was 17. The measurements were repeated in triplicate, and the average value of each was evaluated. The standard acceptable ranges for the Z-average, zeta potential, and PDI are 100–200 nm, ±30 to 40 (mV), and 0.0–0.03, respectively.

### 4.7. Residual Solvent Determination with Gas Chromatography-Mass Spectrometry (GC-MS)

For determination of acetone, Shimadzu GCMS-QP2010 SE (Shimadzu Europa GmbH, Duisburg, Germany) gas chromatography equipment with a 30 m long, 0.25-mm-diameter ZBWax-Plus column using He as the carrier gas was applied. The instrument was calibrated with a five-point series for acetone.

### 4.8. Encapsulation Efficiency, Percentage Yield, and Drug-Loading Determination

To measure the encapsulation, percentage yield, and drug loading of PG-loaded liposomes, the centrifuge method was used. The nanoparticles were first centrifuged for 1 h at 22,413 relative centrifugal force (RCF 16,500 rpm, 4 °C) in a Hermle Z323 laboratory centrifuge. The supernatant was collected and screened by using Agilent 1260 (Agent Technologies, Santa Clara, CA, USA) HPLC. A Gemini-NX^®^ C18 column (5 µm, 150 mm × 4.6 mm; Phenomenex, Torrance, CA, USA) was used as a stationary phase. The mobile phase was purified water adjusted to pH = 3.0, with phosphoric acid and acetonitrile in an 80:20 ratio. Twenty microliters of each sample was injected. Separation was performed by 10 min isocratic elution at 25 °C. The eluent flow rate was 1 mL/min, and the chromatograms were detected at 254 nm using a UV-VIS diode-array detector. For evaluation of data, ChemStationB.04.03 software (Agilent Technologies, Santa Clara, CA, USA) was used. The retention time of PG was 4 min. The linear regression of the calibration line was 0.998. The limit of detection (LOD) and limit of quantification (LOQ) in the case of PG were 21 ppm and 63 ppm, respectively. Each sample was run in triplicate, and the percentage of drug encapsulated was calculated by using the following formula [47]:

(4) $$\mathrm{Encapsulation}\;\mathrm{Efficiency}\;\left(\%\right)=\frac{w_{1}-w_{2}}{w_{1}}\times 100$$

where w_1_ is the total PG in the liposomes and w_2_ is the free PG in the supernatant.

The percentage yield was calculated by weighing the lyophilized liposomes and evaluated by using the following formula:

(5) $$\mathrm{Percentage}\;\mathrm{Yield}\;\left(\%\right)=\frac{\mathrm{Actual}\;\mathrm{Yield}}{\mathrm{Theoretical}\;\mathrm{Yield}}\times 100$$

Drug loading was calculated by weighing the lyophilized liposomes and evaluated by using the following formula:

(6) $$\mathrm{Drug}\;\mathrm{loading}\;\left(\%\right)=\frac{w_{1}-w_{2}}{w_{NP}}\times 100$$

where w_1_ is the amount total PG in the liposomes, w_2_ is the free PG in the supernatant, and w_NP_ is the amount of formulation used for analysis.

### 4.9. Fourier-Transform Infrared Spectroscopy (FTIR)

The FTIR spectra of pure PG, cholesterol, phospholipids, and coating material were collected using a Thermo Nicolet AVATAR FTIR spectrometer (Thermo-Fisher, Waltham, USA) in the spectral range of 4000 and 400 1/cm, with an optical resolution of 4 1/cm. The sample was mixed with 150 mg of dry KBr and compressed to prepare the pellet.

### 4.10. Differential Scanning Calorimetry (DSC)

To investigate the physiochemical properties and changes to analyze the crystallinity of solid-state products, DSC measurements were carried out. In this evaluation, HA-coated liposomes were investigated to assess the possible intermolecular interactions between the drug, the HA-coated material, and lipids. DSC measurements were performed using a Mettler Toledo DSC 821e instrument (Mettler-Toledo GmbH, Greifensee, Switzerland). Approximately 3–5 mg of samples of physical mixtures, all components used in formulation development along with the coated material and product samples, were loaded into an aluminum pan and examined in the scanning temperature range of 25–300 °C, with an empty Al pan used as reference. The heating rate was 20 °C/min in the presence of argon as a carrier gas with a flow rate of 150 mL/min. The data analysis of the components was performed by using STARe software V9.0 (Mettler-Toledo GmbH, Greifensee, Switzerland). Each measurement was normalized to the sample size.

### 4.11. X-ray Powder Diffraction (XRPD)

The XRPD method was used for structural characterization to investigate the effect of the preparation procedure on the particle characteristics, to check the state of the API entrapped in the liposomes, as well as to confirm the lack of lipid–drug interaction. The diffractograms of pure PG, coated liposomes, and physical mixture were obtained by using a BRUKER D8 Advance X-ray powder diffractometer (Bruker AXS GmbH, Karlsruhe, Germany). All the results were obtained with a slit-detector Cu Kλ1 radiation (λ = 1.5406 Å) source. This study was also helpful in analyzing the amorphous and crystalline structure of the drug compound and to predict in vitro release behavior. All the components of the formulation and the coated formulation itself were analyzed and scanned at 40 kV and 40 mA in the angular range of 3°–40° 2θ at a step time of 0.1 s and a step size of 0.007°. The samples were analyzed in a quartz holder and measured at ambient temperature and humidity.

### 4.12. Surface Morphology

The liposome formulations were screened to check the surface properties via transmission electron microscopy (TEM) (FEI Tecnai G2 20 X Twin; FEI Corporate Headquarters, Hillsboro, OR, USA) operated at a 200 kV accelerating voltage. A few microliters of the liposome dispersion was put onto a carbon-coated copper grid; filter paper was used to remove the extra drops of the sample suspension from the grid, leaving a thin liquid film spread over the holes. The sample was negatively stained with sodium silicotung-state solution directly within 1 min of being deposited. Filter paper was used to remove excess sodium silicotung-state solution, and the stained samples were observed [48].

### 4.13. In Vitro Release Test

The release study was performed under nasal conditions at 35 °C by using the dialysis method under constant stirring at 50 rpm. In a dialysis sac, 5 mg of lyophilized formulation was placed, and this was placed in 50 mL of simulated nasal electrolyte solution (SNES), which combined 8.77 g of NaCl, 2.98 g of KCl, and 0.59 g of CaCl_2_ anhydrous in 1000 mL of deionized water at pH 5.60 [37]. The samples were withdrawn at predetermined time intervals for up to 120 h. After filtration of the withdrawn volume, the drug concentration was determined by the HPLC method described above. All the measurements were repeated in triplicate. The in vitro release data of the samples were analyzed kinetically by fitting mathematical models (first order, zero order, Higuchi, and Korsmeyer–Peppas model). The results of the release study were evaluated.

### 4.14. In Vitro Permeability Study

In vitro permeability studies were performed on a modified horizontal side-by-side diffusion apparatus at 35 °C under 100 rpm continuous stirring (Thermo Haake C 10-P5, Sigma-Aldrich Co.). The two compartments were separated by an isopropyl-myristate-impregnated cellulose membrane (Pall Metricel cellulose membrane, 0.45 μm pore size). The volumes of the donor and acceptor phases were both 9 mL, with a 0.69 cm^2^ diffusion surface. The donor phase was pH = 5.6 SNES, and the acceptor phase was pH = 7.4 phosphate buffer saline solution (PBS). In the donor phase, 12 mg of the PG-containing formulation was inserted, and 2 mL of samples were withdrawn from the acceptor phase and replaced with fresh acceptor medium at predetermined time intervals (5, 10, 15, and 60 min) during the investigation. The concentration of the drug diffused via the membrane was determined by the HPLC method described above; each sample was analyzed in triplicate.

### 4.15. Hydrogen Peroxide (H_2_O_2_)-Scavenging Assay

A solution of hydrogen peroxide (40 mM) was prepared in 0.05 M phosphate buffer (pH 7.4). PG liposomes with different drug concentrations (125, 250, and 500 μg/mL) were added to the hydrogen peroxide solution (0.6 mL, 40 mM). The absorbance of hydrogen peroxide at 230 nm was determined after 10 min against a blank solution containing phosphate buffer without hydrogen peroxide. The percentage hydrogen-peroxide-scavenging activity was then calculated using the following equation:

(7) $$H_{2}O_{2\;}scavanging\;effect\;\left(\%\right)=A_{0}-\frac{A}{A_{0}}\times 100$$

where *A*_0_ is the absorbance of the control reaction and *A* is the absorbance in the presence of the initial PG-containing sample.

### 4.16. Stability Studies of Coated and Uncoated Liposomes

Stability studies were carried out to ensure the efficacy of the formulations throughout the shelf life. Accelerated stability studies were performed in accordance with ICH guidelines (Q1A R2), and the effect of temperature (40 ± 2 °C) and relative humidity (75 ± 5%) on formulation stability was observed for both coated and uncoated liposomal formulations for a duration of 6 months (at 0, 1, 3, and 6 months). All formulations (coated and uncoated liposomes) were evaluated for physical appearance, Z-average, PDI, zeta potential, and EE. The chemical stability of the drug was investigated retrospectively. The drug content was determined with the HPLC method described above after 2, 5, and 8 months of storage, and the shelf life (t_90%_) was extrapolated.

### 4.17. Statistical Analysis

The statistical analysis of the results of this research data was performed using Microsoft^®^ Excel (Microsoft Office Professional Plus 2013) and JMP^®^ 13 software (SAS Institute, Cary, CA, USA). All the results were replicated in triplicate and presented with mean and standard deviation. In vitro release and in vitro permeability data were processed by using one-way analysis of variance (ANOVA). For the in vitro scavenging assay, a *t*-test was performed. Differences were considered significant when *p* < 0.05.

## 5. Conclusions and Future Perspectives

The DPM as a novel liposome preparation method has not been previously applied for the preparation of liposomes. As a gentle formulation method, it is suitable for loading antioxidants into liposomes, preserving their stability. Regulatory authorities suggest the application of the QbD approach for designing an optimized product suitable for intranasal delivery. This study helped in the preparation of coated liposomes, and PG was used as the model compound that has proven anti-cancer efficacy. It is expected to be applied in co-encapsulation with other chemotherapeutic drugs in the future and will help to target glioblastoma multiforme via the intranasal route. The results demonstrated that the prepared liposomes were stable even under accelerated conditions. The in vitro scavenging, release and permeation study results showed significant potential of PG liposomes for intranasal delivery.

## Figures and Tables

**Table 1:** Selection of quality target product profiles (QTPPs) of PG-loaded liposomes for nose-to-brain delivery, along with respective target and justification.

QTPP	Target	Justification
Therapeutic effect	The CNS (the model API is PG)	Circumvention of the blood–brain barrier (BBB). PG has antioxidant and anti-inflammatory activity that are essential in the treatment of cancer [26].
Administration route	Nasal administration (nose-to-brain delivery)	Non-invasive, direct, and more effective route of administration than other invasive routes. Nanoparticles are transported from endothelial cells to the olfactory neurons via endocytosis or pinocytosis and move along the axon or follow the trigeminal nerve pathway [27,28].
Dosage form	Liquid nano-formulation	Enhanced biocompatibility, high scalability, safety, and efficacy of lipid nanoparticles. A liquid formulation is more feasible for nose-to-brain administration [29].
Coated lipid nanoparticles	Enhanced stability	The hyaluronic acid (HA) coating will enhance the stay time within the nasal mucosa. Active target delivery at the CD44 receptor is possible via coating with HA. Additionally, it will synergize stability [30].
Targeting pathway	Preferred particle size range 100–700 nm	Nanoparticles within the range of 20–200 nm can follow clathrin-coated pits, and nanoparticles in the size range of 200–1000 nm can be transported via caveolae-mediated endocytosis.

**Table 2:** Z-average, polydispersity index (PDI), and zeta potential of 15 runs on the design of experiments.

Number of Runs	Temperature (°C)	Amount of Phospholipids (mg)	Amount of Cholesterol (mg)	Z-Average (nm) *	PDI *	Zeta Potential (mV) *
1	50	16	16	150 ± 10	0.27 ± 0.01	−22 ± 8.4
2	70	16	16	155 ± 5.5	0.28 ± 0.02	−18 ± 6.5
3	50	32	16	145 ± 4.5	0.29 ± 0.02	−23 ± 8.4
4	70	32	16	140 ± 5.5	0.28 ± 0.05	−24 ± 8.4
5	50	24	8	125 ± 6.6	0.25 ± 0.07	−27 ± 7.5
6	70	24	8	125 ± 7.8	0.22 ± 0.01	−28 ± 8.5
7	50	24	24	400 ± 8.8	0.40 ± 0.08	−8 ± 10.2
8	70	24	24	450 ± 22	0.45 ± 0.09	−7 ± 12
9	60	16	8	121 ± 2.3	0.24 ± 0.08	−33 ± 5.5
10	70	32	8	123 ± 2.4	0.25 ± 0.06	−28 ± 6.5
11	60	16	24	430 ± 40	0.55 ± 0.08	−6 ± 10
12	60	32	24	420 ± 20	0.49 ± 0.05	−8 ± 10.2
13	60	24	16	130 ± 12	0.21 ± 0.01	−29 ± 3.3
14	80	24	16	135 ± 10	0.22 ± 0.02	−26 ± 5.5
15	70	24	16	142 ± 8	0.27 ± 0.02	−25 ± 6.2

* Data are the mean ± SD (*n* = 3 independent formulations).

**Table 3:** Analysis of particle size, PDI, and zeta potential of uncoated and coated liposomes.

Formulation	Z-Average * (nm)	PDI *	Zeta Potential * (mV)
Uncoated liposomes	135.2 ± 5.2	0.094 ± 0.001	−29.9 ± 5.8
Coated liposomes	167.9 ± 3.5	0.129 ± 0.002	−33.6 ± 4.5

* Data are the mean ± SD (*n* = 3 independent formulations).

**Table 4:** Concentration of residual organic solvent in the optimized formulations.

Formulation	Acetone (ppm)	Maximum Residual Level * (ppm)
Uncoated liposomes	442 ppm	5000
Coated liposomes	47 ppm	

* Based on the International Conference for Harmonisation (ICH) Q3C (R5).

**Table 5:** R^2^ values of drug release from uncoated and HA-coated liposomes.

Kinetic Model	Zero Order	First Order	Korsmeyer–Peppas	Higuchi	Hixson–Crowel
R^2^ value of uncoated liposomes	0.7211	0.8922	0.8656	0.9238	0.8691
R^2^ value of coated liposomes	0.745	0.8935	0.8662	0.9073	0.8488

**Table 6:** Accelerated stability studies of coated and uncoated liposomes.

Formulation	Time (Months)	Decrease in EE * (%)	Z-Average * (nm)	PDI *	Z.P. * (mV)	P.A.
Uncoated liposomes	0 3 6	N.C. 10 ± 2.0 30 ± 3.0	N.C. N.C. 550 ± 10	0.094 ± 0.03 0.095 ± 0.01 0.511 ± 0.54	−29.9 ± 2.3 −27.7 ± 3.3 −15.6 ± 4.5	Milky dispersion Milky dispersion Milky dispersion
Coated liposomes	0 3 6	N.C. N.C. 5 ± 2	N.C. ~170.2 ± 5.5 180 ± 7.6	N.C. 0.130 ± 0.02 0.222 ± 0.03	−33.6 ± 3.5 −29.9 ± 5.5 −27.7 ± 6.4	Milky dispersion Milky dispersion Milky dispersion

* Data are the mean ± SD (*n* = 3 independent formulations); * P.A., physical appearance; * Z.P., zeta potential; * EE, encapsulation efficiency; N.C., no change.

## Data Availability

The data presented in this study are available on request from the corresponding author.

## Sample Availability

Samples of the compounds are not available from the authors.
